# Comparisons between minimally invasive and open esophagectomy for esophageal cancer with cervical anastomosis: a retrospective study

**DOI:** 10.1186/s13019-020-01182-3

**Published:** 2020-06-08

**Authors:** Zongjie Li, Canhui Liu, Yuanguo Liu, Sheng Yao, Biao Xu, Guohua Dong

**Affiliations:** Department of Thoracic and Cardiovascular Surgery, The Third Affiliated Hospital of Nanjing University of Chinese Medicine, Nanjing Municipal Hospital of Traditional Chinese Medicine, Nanjing, 210001 Jiangsu Province China

**Keywords:** Minimally invasive esophagectomy, Open esophagectomy, Esophageal carcinoma, Cervical anastomosis, Retrospective analysis

## Abstract

**Background:**

As an extensive surgery, minimally invasive esophagectomy (MIE) has advantages in reducing morbidity and improving quality of life for patients suffering from esophageal cancer. This study aims to investigate differences between MIE and open esophagectomy (OE) for considerations of the safety of procedures, rate of tumor resection, postoperative complications, and quality of life. This paper also tends to provide some references for MIE on esophageal cancer therapy.

**Methods:**

A retrospective data analysis was undertaken on 140 patients who either underwent MIE or OE for esophageal cancer with cervical anastomosis from March 2013 to May 2014 by our surgical team. Preoperative characteristics were analyzed for both groups. Differences in perioperative and oncologic outcomes were compared in operation time, intraoperative blood loss, lymph nodes retrieved, and R0-resection rate. Accordingly, a comparative analysis was conducted on complications namely anastomotic leakage, pulmonary infection, in-hospital mortality, and short-term (3 months) postoperative EORTC C30 Global health as well.

**Results:**

A total of 140 patients (87 with MIE and 53 with OE) were enrolled and the two groups were homogeneous in terms of patient- and tumor-related data. There was no difference on postoperative ICU stay (21.15 ± 1.54 h vs 21.75 ± 1.68 h, *p* = 0.07) and R0-resection rate (100% vs 100%, *p* = 1.00). The operation time for MIE was significantly shorter (146.08 ± 17.35 min vs 200.34 ± 14.51 min, *p* <  0.0001), the intraoperative blood loss was remarkably saved (MIE vs OE, 83.91 ± 24.72 ml vs 174.53 ± 35.32 ml, *P* <  0.0001) and more lymph nodes were retrieved (MIE vs OE, 38.89 ± 4.31 vs 18.42 ± 3.66, *P* <  0.0001). There was no difference between the groups to postoperative complications and mortality. However, pulmonary infection in MIE was higher than in OE and the difference was not statistically significant (MIE vs OE, 20.75% vs 31.03%, *P* = 0.24). Complications such as in-hospital mortality and short-term (3 months) postoperative EORTC C30 Global health displayed no difference between both groups as well.

**Conclusions:**

The number of lymph nodes and intraoperative blood loss were significantly ameliorated in MIE. A 4–5 cm longitudinal incision below the xiphoid process was made to create the gastric conduit under direct vision assisting in shortening the total operation time significantly.

## Background

Esophageal carcinoma is a kind of digestive system tumor with high malignancy and poor prognosis. It has been reported that the overall survival rate of esophageal cancer in 5 years is only 15–20% [1], which turns into the sixth leading cause of cancer-related death worldwide [2]. Squamous cell carcinoma (SCC) and adenocarcinoma (AC) present to be the most common pathological types of esophageal cancer, among which squamous cell carcinoma develops as a principal pathological type. The distribution of esophageal cancer demonstrates significant regional differences [3]. The incidence of SCC mostly occurred in Asia, especially in China (more than half of global SCC cases), while the highest burden of AC was found in Western countries [4]. The incidence of AC in the Western world has been a marked and steady increase over recent decades which takes predominantly in esophagectomy patients [5]. Current treatment strategies for esophageal cancer have developed into two main directions: local-regional therapy and systemic treatment. At the same time, optimized individual therapeutic protocol should be achieved for patients with esophageal cancer based on the type of tumor, location, local infiltration, and individual physiological conditions. However, esophagectomy with radical lymph nodes dissection is recognized as one of the standard treatments for patients with localized and locally advanced disease [6]. Due to the complexity of surgical procedures, huge trauma, severe postoperative complications (especially pulmonary infection), and poor quality of life after operation [7], surgical treatment still has risks in a high mortality rate andother disadvantages as well, along with advances in both medical technology and postoperative care.

As medical technologies advance rapidly, extensive attention and application of video-assisted thoracoscopic surgery with trauma reduction have gainedfrom thoracic surgeons, especially in China [8]. Numerous comparative studies on MIE and OE have revealed that MIE has produced satisfactory results in lower rate of postoperative complications, better relieved pain, less blood loss, more lymph nodes retrieved, shorter hospital stay, better postoperative short-term quality of life and lower overall mortality rates [9, 10]. However, no consensus has been reached on the indications for MIE treatment yet, which is generally determined by the surgeon’s clinical experience currently. More comparative studies between MIE and OE have been carried out focusing on safety of procedures, rate of the tumor resection, postoperative survival rate, and quality of life.

Data associated with MIE and OE has been obtained for the treatment of middle or upper esophageal carcinoma in this study. Analysis on the safety of procedures, rate of tumor resection, postoperative complications and quality of life has been performed in order to provide relevant references for MIE on esophageal cancer therapy.

## Methods

### Patient selection

A total of 140 patients (87 MIE and 53 OE) underwent surgeries for esophageal cancer were enrolled from March 2013 to May 2014 by our surgical team. All patients were performed preoperative histopathologic diagnoses of esophageal cancer by gastroscopy and pathological display. Moreover, locations of tumors were noticed in the middle or upper segment of the esophagus. By preoperative chest preparation and upper abdominal enhanced CT examination, the size of the tumor and the extent of surrounding lymph nodes invasion were assessedfor eliminating metastasis of distant tissues and organs. The study was approved by the Ethics Committee of The Third Affiliated Hospital of Nanjing University of Chinese Medicine.

### Surgical approach

All sugeries in the two groups were performed by our surgical team and all patients underwent resection of the esophageal tumor, lymph nodes dissection, jejunostomy, and left neck anastomosis.

MIE: (1) Thoracic portion. The patient was positioned in a left lateral decubitus for the thoracic portion. A standard right video-assisted thoracoscopic surgery (VATS) 4-trocar approach with CO_2_ insufflation at 8 mmHg was implemented. Both ends of the azygos veins were closed with Hemolok and cut with an ultrasonic scalpel. Then apply ultrasonic scalpel complete resection of esophagus along the thoracic esophageal bed from the top of the right thoracic cavity to the hiatus of esophageal diaphragm, lymph nodes were dissected routinely (posterior superior vena cava, right recurrent laryngeal nerve, left-right recurrent laryngeal nerve, carina, paraesophageal, inferior pulmonary vein, and cardiac side), finally place a drainage tube and sew up the incisions. (2) Abdominal portion. The patient was positioned with a steep reverse Trendelenburg position. A standard laparoscopic 5-trocar approach with CO_2_ insufflation at 13 mmHg was implemented. The left gastric arteriovenous and partial of the short gastric artery were interrupted sequentially with ultrasonic scalpel, starting from the lesser curvature of stomach to fundus ventriculi. Lymph nodes were dissected in a routine manner (common hepatic artery, left gastric arteriovenous, and lesser curvature). Then, the omentum was opened between the stomach and colon transversum. Next, the left gastroepiploic artery and partial short gastric arteries were dissected and the operation directed to fundus until linked up with the lesser curvature of the stomach. It was worth noting that the right gastroepiploic artery arch should be well protected during the surgical processes. Dissect along the greater curvature to pylorus until the stomach was dissociated completely. Finally, laparoscopic jejunostomy was used for nutrition supply postoperatively and the instruments for minimally invasive surgery were removed. A 4-5 cm longitudinal incision was made below the xiphoid process to expose the stomach and a 4- to 5-cm-wide gastric conduit was made by multiple firings of a linear stapler. At last, the gastric conduit was retracted into the abdominal cavity and the incisions were sutured. (3) Cervical portion. A 3 cm transverse incision along the direction of dermatoglyph was performed in the left lateral cervical part, the organ was visible and the anastomosis was accomplished.

OE: Standard three incisions were performed including: right thoracic incision, median abdominal incision and left cervical incision. Operation in the thoracic portion was implemented at the right thorax along the fifth intercostal posterolateral 15–20 cm incision. Surgical procedures and methods of lymph nodes resection were similar to those of MIE. Operation of the abdominal portion was carried out from the xiphoid to the umbilical abdominal median incision and remaining procedures were similar to MIE as well. In the end, the jejunostomy was performed under direct vision. The operation of the cervical portion was the same as MIE.

### Statistical analysis

SPSS 18.0 software was adopted for data analysis (IBM, Armonk, NY). Continuous variables were expressed as mean ± standard deviation (SD). To evaluate differences between the groups, Chi-square (or Fisher exact) test was adopted for binomial variables. Mann-Whitney test was used for continuous variables that did not meet a normal distribution. A *p-*value of less than 0.05 was taken as a level of significance for all analyses.

## Results

### Demographic clinical characteristics

Between March 2013 and May 2014, a total of 140 patients with middle or upper esophageal cancer were performed surgeries by our surgical team: 53 OE and 87 MIE. Majority of patients were male (77.14%). The median age in MIE was 64.75 ± 7.80 years old and 63.02 ± 6.84 in OE, which revealed no difference (*P =* 0.24). Body mass index (BMI) and the percentage of patients who had a history of smoking were also similar between two groups. The remaining indicators listed in the table displayed no statistically significant difference as well (Table 1).

**Table 1:** Preoperative Characteristics of Patient and Tumor

Characteristics	OE (*n* = 53)	MIE (*n* = 87)	*P*-Value
Gender (%)			0.53^2^
Male	73.58%	79.31%	
Female	26.42%	20.69%	
Age (years)	63.02 ± 6.84	64.75 ± 7.80	0.24^1^
BMI (kg/m^2^)	21.51 ± 1.56	21.75 ± 1.53	0.42^1^
ASA grade (%)			0.12^2^
I	35.85%	27.59%	
II	50.94%	48.28%	
III	13.21%	24.13%	
Smoking history (%)	67.92%	74.71%	0.44^2^
Tumor stage (%)			0.66^2^
T1	12%	21%	
T2	68%	54%	
T3	20%	25%	
Comorbidity (%)			0.46^2^
Hypertension	50.94%	74.71%	
Diabetes	9.4%	13.79%	
Coronary artery disease	15.09%	24.14%	
Cardiac arrhythmia	5.66%	10.34%	
Pulmonary disease	33.96%	35.63%	
History of stroke	9.43%	11.49%	
Neoadjuvant therapy (%)	5.66%	8.05%	0.74^2^

Scale variables were expressed as median and range, ordinal and nominal parameters as absolute numbers, and percent

*ASA* American Society of Anesthesiologists, *BMI* Body mass index.

^1^Mann-Whitney test

^2^Chi-square (or Fisher exact) test.

### Operative and oncologic outcomes

Perioperative and oncologic outcomes were shown in Table 2. The operation time was 146.08 ± 17.35 min in MIE group and 200.34 ± 14.51 min) in OE (*P* <  0.0001). The intraoperative blood loss in MIE was significantly decreased compared with OE (83.91 ± 24.72 ml vs 174.53 ± 35.32 ml, *P* <  0.0001). Notably, there were more lymph nodes retrieved in MIE compared to OE (38.89 ± 4.31 vs 18.42 ± 3.66, *P* <  0.0001). Moreover, the postoperative hospital stay in MIE was shorter compared with OE (9.68 ± 2.97 days vs 15.64 ± 6.05 days, *P* <  0.0001). Other outcomes such as blood transfusion rate, postoperative ICU stay, R0-resection rate, and postoperative pathological staging were similar between the groups.

**Table 2:** Perioperative and oncologic outcomes

Variable	OE (*n* = 53)	MIE (*n* = 87)	*P*-Value
Operation time (min)	200.34 ± 14.51	146.08 ± 17.35	< 0.0001^1^
Intraoperative blood loss (mL)	174.53 ± 35.32	83.91 ± 24.72	< 0.0001^1^
Blood transfusion (%)	3.77%-2/53	2.30%-2/87	0.63^2^
Postoperative ICU stay (hours)	21.75 ± 1.68	21.15 ± 1.54	0.07^1^
Postoperative hospital stay (days)	15.64 ± 6.05	9.68 ± 2.97	< 0.0001^1^
R0-resection rate (%)	100%-53/53	100%-87/87	1.00^2^
Lymph nodes retrieved	18.42 ± 3.66	38.89 ± 4.31	< 0.0001^1^
Postoperative pathological staging			0.28^2^
I	22.64%	21.83%	
II	58.49%	47.13%	
III	18.87%	31.03%	

Scale variables were expressed as median and range, ordinal and nominal parameters as absolute numbers, and percent

*ICU* Intensive care unit

^1^Mann-Whitney test

^2^Chi-square (or Fisher exact) test

### Postoperative complications and mortality

Postoperative complications and mortality were shown in Table 3. The incidence of anastomotic leakage in MIE was slightly higher compared with OE (8.05% vs 5.66%, *P =* 0.74). Moreover, the risk of pulmonary infection was also higher in MIE (24% vs 19%, *P =* 0.685), Disappointly, the results did not show a statistical significance. The rate of intestinal obstruction in both groups exhibited no significant difference (3.77% vs 1.15%, *P =* 0.56). All incomplete intestinal obstruction were cured by conservative treatments. Both groups were found equivalent to atrial fibrillation, vocal cord paralysis, regurgitation, chylothorax, and in-hospital mortality (*P* > 0.05). Furthermore, No patient had complications including diaphragmatic hernia and wound infection in MIE.

**Table 3:** Postoperative complications and mortality

Variable	OE (*n* = 53)	MIE (*n* = 87)	*P*-Value
Anastomotic leakage (%)	5.66%	8.05%	0.74^2^
Pulmonary infection (%)	20.75%	31.03%	0.24^2^
Atrial fibrillation (%)	16.98%	21.84%	0.52^2^
Vocal cord paralysis (%)	0%	3.45%	0.28^2^
Regurgitation (%)	26.42%	29.89%	0.70^2^
Chylothorax (%)	3.77%	1.15%	0.56^2^
Intestinal obstruction (%)	3.77%	1.15%	0.56^2^
Diaphragmatic hernia (%)	0%	0%	1.00^2^
Wound infection (%)	3.77%	0%	0.14^2^
In-hospital mortality (%)	1.75%	1.15%	1.00^2^

Scale variables were expressed as median and range, ordinal and nominal parameters as absolute numbers, and percent. ^2^Chi-square (or Fisher exact) test.

Short-term (3 months) postoperative outcomes were shown in Table 4. The incidence of anastomotic stenosis (postoperative 3 months) was low in both groups (24% vs 19%, *P =* 0.685). However, the rate of regurgitation was as high as 29.89% in MIE and 26.42% in OE (*P =* 0.70). Patients with pneumonia in OE were fewer than those in MIE (20.75% vs 31.03%, *P =* 0.24) indicating no marked statistical difference. In addition, complications namely the risk of recurrence and mortality revealed no significant difference (*P* > 0.05). The short-term (3 months) EORTC C30 Global health in MIE was superior to OE, whileno significant difference exhibited between the two groups as well (66.42 ± 5.92 vs 66.10 ± 5.43, *P =* 0.05).

**Table 4:** Short-term (3 months) postoperative outcomes on morbidity, mortality and quality of life

Variable	OE (*n* = 52)	MIE (*n* = 86)	*P*-Value
Stenosis anastomosis	1.92%	2.33%	1.00^2^
Regurgitation	15.38%	23.26%	0.65^2^
Pneumonia	3.77%	8.14%	0.48^2^
Recurrence	0%	0%	1.00^2^
Mortality	0%	3.49%	0.29^2^
EORTC C30 Global health	66.10 ± 5.43	66.42 ± 5.92	0.05^1^

Scale variables were expressed as median and range, ordinal and nominal parameters as absolute numbers, and percent. ^1^Mann-Whitney test; ^2^Chi-square (or Fisher exact) test EORTC: European Organization for Research and Treatment of Cancer Quality of Life Questionnaires. EORTC C30 Global health: scores range from 0 to 100, with higher scores representing better well-being.

## Discussion

For patients with esophageal cancer, surgical resection with the combination of neoadjuvant chemoradiotherapy or chemotherapy was considered as the only potential path for a radical cure currently [11, 12]. However, patients underwent OE were extremely traumatic and suffered from severe postoperative complications, including pulmonary infection and poor quality of life [7]. Since 1992 Cuschieri et al have reported [13] the first application of minimally invasive technique adopted for resection of esophageal cancer. As it produced great potential in minimizing invasions and accelerating rehabilitation, the application of this technique gained popularity in the medical front. However, many scholars hold a conservative attitude on MIE including the complexity of the procedure, adequacy of resection and nodal clearance in upper third tumors, and availability of MIE in patients who performed chemoradiotherapy [14]. As science and technology advance over the past 30 years, technologyies including high-definition imaging, novel energy devices, and enhanced stapling had been widely used in modern medicine. Meanwhile, a variety of modified MIE procedures had been widely implemented in major medical centers all over the world. No consensus has been reached on whether MIE tends to be superior to OE or notamong centers. However, many comparative studies on the clinical effects of MIE and OE, namely the clinical randomized controlled researches [10], Meta analyses [15] and retrospective studies [16], confirmed that there were no significant differences in both techniques. Contrary to general expectations, results in comparing MIE with OE had shown that MIE was associated with lower operative blood loss, shorter ICUand hospital stays, fewer postoperative respiratory complications, better relieved pain and short-term postoperative quality of life [17]. The outcome of postoperative hospital stay in MIE was significantly shorter than in OE (9.68 ± 2.97 vs 15.64 ± 6.05, *P* < 0.0001) in our study. A growing number of clinical trials of esophagectomy showed that MIE could shorten hospital stay [18–20].

A natural channel for early lymphatic returns and distant skip metastases were provided due to the extensive submucosal lymphatic plexus under the esophageal wall (i.e., lymph nodes adjacent to the primary tumor are not affected, but more distant-located lymph nodes contain metastases) [21]. Thus, the early metastases of lymph nodes were one of the characteristics of esophageal cancer. Studies had reported that about 20–40% of patients with submucosal esophageal cancer developed local lymph node metastasis [22, 23]. Consequently, extensive lymph nodes dissection can maximize the clearance of malignant lymph nodes which played an essential role in inhibiting tumor recurrence and facilicating a long survival time for patients with esophageal cancer. A retrospective study on 3572 cases of esophageal carcinoma from Worldwide Esophageal Cancer Collaboration Group (WECC) found that, patients with complete tumor R0 resection, the more lymph nodes extracted, the higher survival rate exhibited in patients. In addition, the total number of lymph nodes was an independent risk factor prognosticating patients’ survivals [24]. In our study, the number of lymph nodes, intraoperative blood loss, and operative time were significantly ameliorated in MIE when compared with OE. Research by Dhamija et al. emphasized that the extent and effect of lymph nodes dissection in MIE were closely linked with learning curve and surgeons’ experiences [25]. Our surgical team initiated the thoracic minimally invasive surgery combined with open surgery for resection of esophageal cancer since July 2012. Total minimally invasive esophagectomy was finally been developed in March 2013. We maintained that the learning curve had a practical impact on the lymph nodes resection (*p* < 0.0001), operation time (*p* < 0.0001) and intraoperative blood loss (*p* < 0.0001) in early stages of MIE. Recently, a clinical comparative study between HMIE (Hybrid minimally invasive esophagectomy) and OE has revealed that there were no significant differences on the number of lymph nodes resection (22 vs. 20, *p* = 0.459) and R0 resection rate (95 vs 93%, *p* = 0.500) [26]. However, the operation time (329 vs 407 min, *p* < 0.001), blood transfusion rate (18 vs 50%, *p* < 0.001), and length of hospital stay (14 vs 18 days, *p* = 0.002) in HMIE were significantly better than those in OE. We made a 4–5 cm longitudinal incision below the xiphoid process to pull out the stomach and a 4- to 5-cm-wide gastric conduit was built, which helped shorten the total operation time significantly with the best record of 88 min. However, both complexity and prolonged operation time of MIE procedures increased when patients were associated with pleural adhesions, enormous tumor mass and preoperative chemoradiotherapy. Patients who underwent OE and MIE for esophageal cancer in the Society of Thoracic Surgeons General Thoracic Surgery Database (STS Database) showed that approximately 50% of patients had preoperative radiation therapy, which contrasted dramatically with only 5.66% (OE) and 8.05% (MIE) of patients by esophagectomy in our study [27]. The procedure duration of OE (312.0 min) and MIE (443.0 min) in the STS Database was also higher than the operation time (OE vs MIE, 200.34 ± 14.51 vs 146.08 ± 17.35 min, *P* < 0.0001) in our study. We speculated that the absence of preoperative radiation therapy probably was the reason for the shortened operation time. The STS Database Task Force reported that most US patients with esophageal cancer had a BMI greater than 25.0 (66.2%, N = 4321) [28], while Chinese patients presented a BMI less than 25.0. Esophageal resection in overweight patients was associated with increased operative time [29]. This also indicated another possibility which significantly influenced outcomes of shorter operative time.

A lot of studies have reported significantly lower pulmonary complications for those who underwent MIE versus OE [17, 18, 30]. Of note, there are several factors linked to pulmonary complications post procedure, including preoperative status, intraoperative details, and postoperative details [31]. In this study, rates of pulmonary infection in MIE were slightly higher than those in OE, although there was no significant difference demonstrated. And that the pulmonary infection rate of patients who underwent MIE (31.3%) was higher than that reported in other studies (17.1%) [18]. One reason may be that the location of the tumor was in the middle or upper segment of the esophagus. This probably enhanced the pain associated with their MIE procedure and produced a negative effect on the incidence of pulmonary complications. Another reason may be that the differences in BMI ranges between Asian and Western populations generating higher pulmonary infection. Mitzman et al. (2018) reported that among patients who were overweight BMI (25.0 to 34.9, accounting for 55% of all patients), there was a lower risk of having any major, pulmonary, infectious, or other complications when compared with BMI 21.5 [29]. In addition, the extensive lymph nodes dissection in MIE (OE vs MIE, 18.42 ± 3.66 vs 38.89 ± 4.31, *P* < 0.0001) may be considered as another cause. Researches discovered that [32, 33] extensive lymph nodes dissection significantly increased postoperative respiratory complications and delayed patients’ rehabilitation. However, contrary opinions proved that extensive lymph nodes dissection neither elevated the risk of postoperative morbidity nor affected the life quality of patients [34, 35]. Therefore, the relationship between the number of lymph nodes resection and postoperative complications may deserve further investigations.

## Conclusion

Results from our retrospective analysis between MIE and OE showed that the clinical outcomes were similar in both procedures. While the number of lymph nodes and intraoperative blood loss were significantly ameliorated in MIE. Moreover, a 4–5 cm longitudinal incision below the xiphoid process was made to create the gastric conduit under direct vision helped to shorten the total operation time significantly.

## Data Availability

All data generated or analyzed during this study are included in this published article.

## Abbreviations

MIE: Minimally invasive esophagectomy
OE: Open esophagectomy
EORTC C30: European Organization for Research and Treatment of Cancer Core Questionnaire
ICU: Intensive Care Unit
CT: Computed tomography
VATS: Video-assisted thoracoscopic surgery
SD: Standard deviation
WECC: Worldwide Esophageal Cancer Collaboration Group
